# Incidental Meckel’s diverticulum during laparoscopic appendectomy: intraoperative decision-making and stapled diverticulectomy in a young adult – a case report

**DOI:** 10.1097/RC9.0000000000001114

**Published:** 2026-10-21

**Authors:** Francisco Gil Quintero, Luis Rafael Chacón Santos, Andrés Felipe Ramírez Carrillo

**Affiliations:** Department of General Surgery, Hospital Militar Central – Universidad Militar Nueva Granada, Bogotá, D.C., Colombia

**Keywords:** appendectomy, case report, incidental findings, laparoscopy, Meckel’s diverticulum, stapled diverticulectomy

## Abstract

**Introduction and importance:**

Meckel’s diverticulum is the most common congenital anomaly of the gastrointestinal tract. Its incidental discovery during an appendectomy or other laparoscopic exploration poses a recurrent intraoperative dilemma: resect it or leave it alone.

**Presentation of case:**

A 21-year-old male presented with a 72-hour history of migratory right iliac fossa pain and suspected acute appendicitis. Laparoscopic appendicectomy was performed. During exploration of the distal ileum, a 4 × 2 cm broad-based Meckel’s diverticulum was identified 130 cm proximal to the ileocecal valve. As the patient met three of four Park criteria (age < 50, male sex, length >2 cm), laparoscopic stapled diverticulectomy was performed with a single perpendicular firing of a 60-mm endoscopic linear stapler.

**Clinical discussion:**

The lifetime complication risk for an unresected Meckel’s diverticulum ranges from 4.2% to 9%; malignancy occurs in approximately 1%–5% of resected specimens. Contemporary propensity-matched analyses and a 2024 systematic review report low morbidity (approximately 5.7%) and no attributable mortality after incidental resection in selected patients. The Park criteria are a risk-stratification tool rather than a definitive indication for resection.

**Conclusion:**

This case illustrates three practical considerations: (i) focused distal-ileum inspection may facilitate the identification of incidental pathology, though not universally accepted; (ii) the Park criteria may support individualized intraoperative decisions as a risk-stratification tool; and (iii) stapled diverticulectomy with a single perpendicular firing was technically feasible and uncomplicated in this patient. These observations should not be generalized as evidence for routine incidental resection.

## Introduction

Meckel’s diverticulum is the most common congenital anomaly of the gastrointestinal tract, with a population prevalence of 0.3–2.9%^[^1–3^]^. It may be identified incidentally during appendectomy or other laparoscopic abdominal explorations. The cumulative lifetime risk of complications is 4.2–9% and decreases with age^[^3,4^]^.

Anatomically, Meckel’s diverticulum arises from persistence of the omphalomesenteric (vitelline) duct and constitutes a true diverticulum containing all three intestinal wall layers, located on the antimesenteric border of the distal ileum, typically within 100 cm of the ileocecal valve. Approximately half of cases contain heterotopic tissue (most commonly gastric mucosa, less frequently pancreatic tissue). When symptomatic, complications include intestinal obstruction, gastrointestinal bleeding from acid-secreting gastric mucosa, diverticulitis, and perforation. Malignant transformation is uncommon, reported in approximately 1–5% of resected specimens, and predominantly involves neuroendocrine tumors^[^5^]^.

HIGHLIGHTS

- Meckel’s diverticulum has a population prevalence of 0.3–2.9%.
- The Park criteria are a risk-stratification tool for intraoperative decisions.
- A 21-year-old man had a 4 × 2 cm broad-based diverticulum found during appendectomy.
- Stapled diverticulectomy with a single firing was feasible and uncomplicated.
- Exploration of the distal ileum is not universally accepted, but it may be considered.

When encountered as an incidental finding, the question of prophylactic resection remains controversial: older series favored observation, whereas contemporary laparoscopic series, a 2024 systematic review, and a propensity-matched ACS-NSQIP analysis report low morbidity and negligible mortality for incidental resection in selected patients^[^1,6^]^. The Park criteria (age <50 years, male sex, length >2 cm, and presence of histologically abnormal or heterotopic tissue) are intended as a risk-stratification tool rather than a definitive indication for resection^[^7^]^. This case report has been reported in accordance with the SCARE 2025 checklist^[^8^]^.

## Case presentation

### Methods statement

This case report was prepared and reported in line with the SCARE 2025 checklist^[^8^]^. The completed SCARE 2025 checklist is included as a Supplemental Digital Content File, available at: https://links.lww.com/IJSCR/A187.

A 21-year-old Hispanic male (BMI 23.5 kg/m^2^) self-presented to the emergency department of a tertiary academic center with a 72-hour history of migratory periumbilical pain radiating to the right iliac fossa, accompanied by chills, hyporexia, and nausea. He had no relevant past medical, surgical, or family history, no allergies, and was on no chronic medications. On admission, he was normotensive (118/72 mmHg; HR 88 bpm; temperature 37.3 °C), with localized peritoneal signs in the right iliac fossa. Hemoglobin was 15.4 g/dL, leucocyte count 9.8 × 10^9^/L and C-reactive protein 84 mg/L. Bedside ultrasound was inconclusive; further imaging was deferred given an Alvarado score of 7. Crohn’s ileitis, perforated peptic ulcer, nephrolithiasis, and symptomatic Meckel’s diverticulitis were excluded clinically^[^4^]^.

After crystalloid resuscitation, piperacillin/tazobactam 4.5 g and mechanical venous thromboembolism prophylaxis, and after informed consent extending to incidental findings, the patient underwent laparoscopic appendectomy under general anesthesia. A three-port technique was used (10-mm umbilical, 10-mm left iliac fossa, 5-mm suprapubic; pneumoperitoneum at 12 mmHg). A suppurative cecal appendix without perforation was identified. The mesoappendix was skeletonized with a bipolar tissue sealer (LigaSure™, Medtronic); the stump was secured with two gold polymer locking clips (Hem-o-lok™ ML, Teleflex) and divided (Fig. 1A).

**Figure 1.**
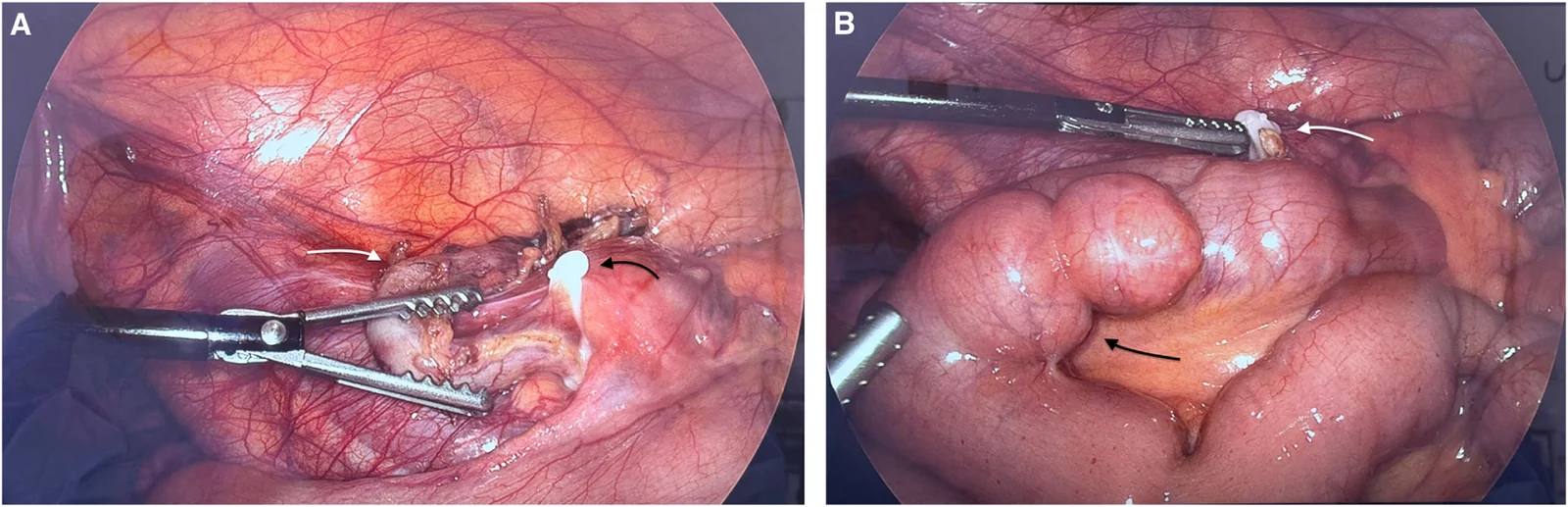
(A) Suppurative cecal appendix (white arrow); a gold polymer locking clip (Hem-o-lok™) at the appendiceal base (black arrow). (B) Broad-based Meckel’s diverticulum on the antimesenteric border of the distal ileum (black arrow); an appendiceal stump with a Hem-o-lok™ clip (white arrow).

Systematic exploration of 200 cm of distal ileum disclosed an asymptomatic broad-based Meckel’s diverticulum measuring 4 × 2 cm, located 130 cm proximal to the ileocecal valve, without macroscopic inflammation or heterotopic tissue (Fig. 1B). Applying the Park criteria as a risk-stratification tool, the patient met three of four criteria (age < 50, male sex, length >2 cm), placing him in a higher-risk subgroup^[^7^]^. Laparoscopic stapled diverticulectomy was performed with a single perpendicular firing of a 60-mm endoscopic linear stapler (Endo GIA™, Medtronic), ensuring a healthy ileal margin and lumen preservation (Fig. 2). The surgeon was board-certified with more than 7 years of advanced laparoscopic experience; operative time was 60 minutes, and blood loss was 10 mL.

**Figure 2.**
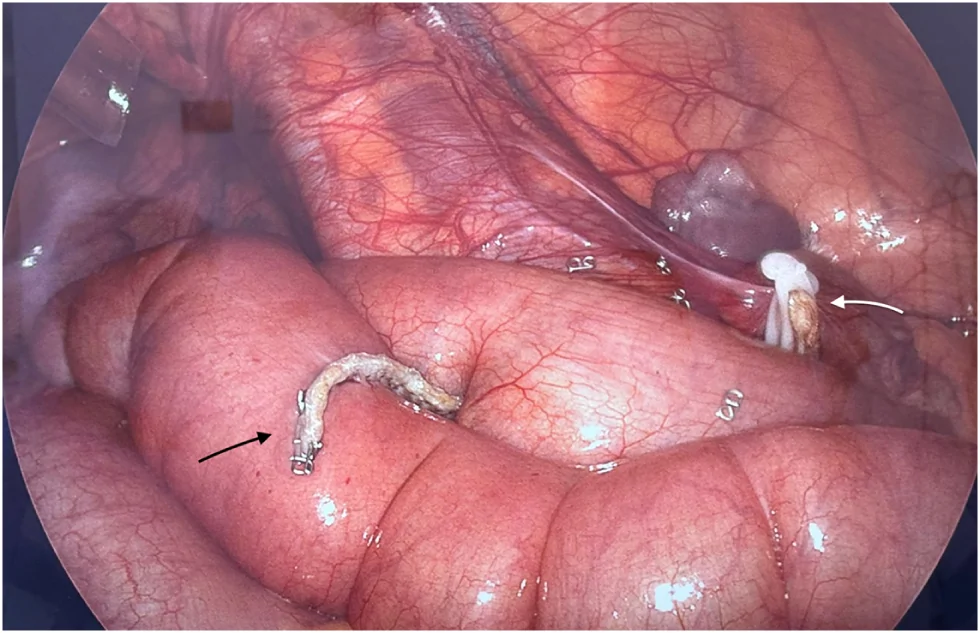
Linear staple line on the antimesenteric ileum after diverticulectomy with a 60-mm endoscopic linear stapler (black arrow) and appendiceal stump with double gold polymer locking clips (white arrow).

The patient tolerated oral intake at 6 hours, ambulated at 12 hours, and was discharged on postoperative day 2. Histopathology confirmed acute suppurative appendicitis and a Meckel’s diverticulum measuring 2 × 2 × 1 cm (post-fixation), without heterotopic mucosa or malignancy. At 3-month telephone follow-up, he was asymptomatic; the postoperative course was Clavien–Dindo grade 0. This short-term follow-up is insufficient to detect delayed complications.

## Discussion

This case illustrates the intraoperative identification of an incidental Meckel’s diverticulum during a laparoscopic appendectomy and its uncomplicated resection using a single-fire perpendicular linear stapler in a single patient.

Routine exploration of the distal-ileum during laparoscopic appendectomy is not universally accepted. Some authors advocate a focused inspection as a low-cost adjunct in experienced centers, whereas others question its added value. We do not advocate systematic exploration as a mandatory step; rather, we present it as a consideration in selected uncomplicated cases when appropriate expertise is available.

The Park criteria constitute a risk-stratification tool derived from the Mayo Clinic series of 1476 patients^[^7^]^ and should not be interpreted as a definitive indication for resection. Contemporary outcome data support resection in selected patients: a 2024 systematic review reported low morbidity (≈5.7%) and no attributable mortality^[^1^]^, and a propensity-matched ACS-NSQIP analysis found no significant differences in 30-day morbidity or mortality between concomitant appendectomy plus diverticulectomy and appendectomy alone^[^6^]^. Malignancy within Meckel’s diverticulum, although uncommon, has also been described and may support resection in selected young patients^[^5^]^.

Interestingly, histopathology did not reveal heterotopic mucosa or malignancy. This highlights a central controversy: although risk-based criteria may support prophylactic resection, some patients undergo resection of lesions that may never have become symptomatic. Intraoperative decision-making should therefore remain individualized and this observation should not be generalized to unselected patients.

From a technical standpoint, laparoscopic stapled diverticulectomy is a commonly used minimally invasive technique for diverticula with a narrow neck or a height-to-diameter ratio greater than 2, and is acceptable for broad-based diverticula when an adequate ileal margin can be obtained and there is no macroscopic suspicion of heterotopic mucosa at the base^[^6,9^]^. Had ectopic gastric mucosa been present, simple diverticulectomy might have been inadequate: surgical best practice would then recommend either a wedge resection extending to the mesenteric border or a formal segmental resection with primary anastomosis to ensure complete excision. Segmental resection is likewise reserved for broad-based diverticula with visible heterotopic mucosa, active hemorrhage, perforation, or suspicion of malignancy.

## Strengths and limitations

Strengths include intraoperative photographic documentation and an explicit decision framework based on the Park risk-stratification tool. **A key limitation is the 3-month follow-up**: although appropriate for detecting immediate complications, it is insufficient to demonstrate long-term benefit of prophylactic resection and cannot rule out delayed complications (staple-line stricture, adhesive obstruction, functional sequelae); longer surveillance would be required. Additional limitations include the single-patient nature of the report and the dependence of the resection decision on the surgeon’s experience. The favorable short-term outcome should not be interpreted as evidence supporting routine incidental resection.

## Patient perspective

During the consented postoperative interview, the patient stated: “I am glad the team explained from the beginning that they could find and treat other problems while operating on my appendix. Knowing that they removed something that might have caused me problems years from now gave me peace of mind.”

## Conclusion

Focused distal ileal inspection during laparoscopic appendectomy may facilitate identification of incidental findings such as Meckel’s diverticulum in selected cases, although this practice is not universally endorsed. In young adults who meet the Park risk-stratification criteria, a single-firing stapled diverticulectomy appeared technically feasible and uncomplicated in this individual case. Practical considerations include: (i) considering focused ileal inspection only in selected uncomplicated appendectomies performed by experienced teams; (ii) using the Park criteria as a risk-stratification tool, not a definitive indication for resection; and (iii) extending preoperative informed consent to cover incidental findings. These observations should not be interpreted as evidence supporting routine prophylactic resection.

## Data Availability

All data relevant to the case have been included in the manuscript. The signed informed consent form and the original clinical records remain confidential and are held by the corresponding author; they can be made available to the Editor on reasonable request.
